# Spatial Coherence Approaches to Distinguish Suspicious Mass Contents in Fundamental and Harmonic Breast Ultrasound Images

**DOI:** 10.1109/TUFFC.2023.3332207

**Published:** 2024-01-09

**Authors:** Arunima Sharma, Eniola Oluyemi, Kelly Myers, Emily Ambinder, Muyinatu A. Lediju Bell

**Affiliations:** Department of Electrical and Computer Engineering, Johns Hopkins University, Baltimore, MD 21218 USA; Department of Radiology and Radiological Science, Johns Hopkins Medicine, Baltimore, MD 21287 USA.; Department of Radiology and Radiological Science, Johns Hopkins Medicine, Baltimore, MD 21287 USA.; Department of Radiology and Radiological Science, Johns Hopkins Medicine, Baltimore, MD 21287 USA.; Department of Electrical and Computer Engineering, the Department of Biomedical Engineering, the Department of Computer Science, and the Department of Oncology, Johns Hopkins University, Baltimore, MD 21218 USA

**Keywords:** Breast imaging, coherence-based beamforming, harmonic imaging, ultrasound

## Abstract

When compared to fundamental B-mode imaging, coherence-based beamforming, and harmonic imaging are independently known to reduce acoustic clutter, distinguish solid from fluid content in indeterminate breast masses, and thereby reduce unnecessary biopsies during a breast cancer diagnosis. However, a systematic investigation of independent and combined coherence beamforming and harmonic imaging approaches is necessary for the clinical deployment of the most optimal approach. Therefore, we compare the performance of fundamental and harmonic images created with short-lag spatial coherence (SLSC), M-weighted SLSC (M-SLSC), SLSC combined with robust principal component analysis with no M-weighting (r-SLSC), and r-SLSC with M-weighting (R-SLSC), relative to traditional fundamental and harmonic B-mode images, when distinguishing solid from fluid breast masses. Raw channel data acquired from 40 total breast masses (28 solid, 7 fluid, 5 mixed) were beamformed and analyzed. The contrast of fluid masses was better with fundamental rather than harmonic coherence imaging, due to the lower spatial coherence within the fluid masses in the fundamental coherence images. Relative to SLSC imaging, M-SLSC, r-SLSC, and R-SLSC imaging provided similar contrast across multiple masses (with the exception of clinically challenging complicated cysts) and minimized the range of generalized contrast-to-noise ratios (gCNRs) of fluid masses, yet required additional computational resources. Among the eight coherence imaging modes compared, fundamental SLSC imaging best identified fluid versus solid breast mass contents, outperforming fundamental and harmonic B-mode imaging. With fundamental SLSC images, the specificity and sensitivity to identify fluid masses using the reader-independent metrics of contrast difference, mean lag one coherence (LOC), and gCNR were 0.86 and 1, 1 and 0.89, and 1 and 1, respectively. Results demonstrate that fundamental SLSC imaging and gCNR (or LOC if no coherence image or background region of interest is introduced) have the greatest potential to impact clinical decisions and improve the diagnostic certainty of breast mass contents. These observations are additionally anticipated to extend to masses in other organs.

## Graphical Abstract

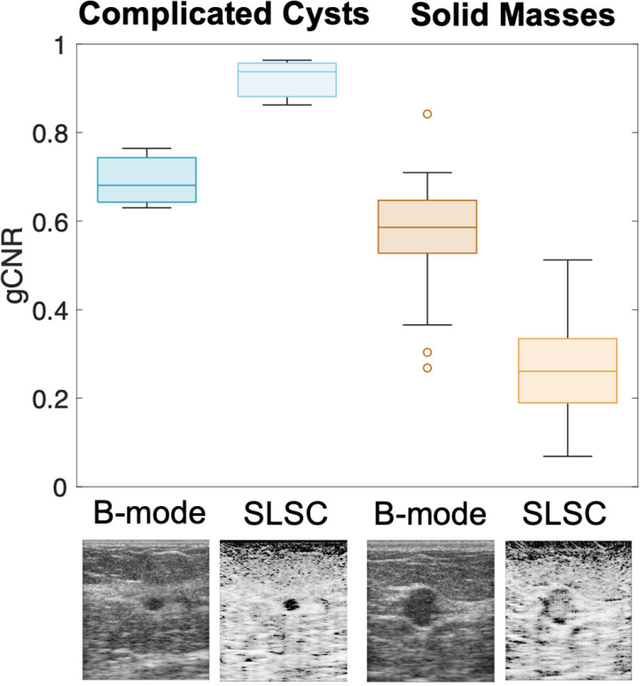

## I. INTRODUCTION

ULTRASOUND imaging is a safe, portable, low-cost, noninvasive imaging modality commonly employed in multiple in vivo applications, including fetal imaging [1], Harmonic imaging is based on the principle of nonlinear propagation of acoustic waves inside biological tissues. kidney stone detection [2], gall bladder imaging [3], breast imaging [4], and suspicious mass detection throughout the body [5]. Although applications of ultrasound imaging continue to expand and advances in image quality are everevolving, the persistent low-contrast of soft tissues, the presence of acoustic clutter, and the similar appearance of different mass types limit even greater usage of ultrasound imaging in biomedical applications [6], [7], [8], [9], [10]. Multiple methods have been implemented to decrease acoustic clutter and improve ultrasound image quality, including harmonic imaging and coherence-based beamforming.

The nonlinear distortion of transmitted waves leads to the generation of ultrasound waves with frequencies that are integer multiples of the transmitted (i.e., fundamental, $f_{0}$) frequency [11]. Typically, resulting waves with 2 $f_{0}$ frequency are utilized to form the harmonic images and the higher harmonics are ignored because of the limited bandwidth of the ultrasound transducer and the low signal-to-noise ratio (SNR) of higher harmonics. When compared to fundamental B-mode imaging, harmonic B-mode imaging improves organ visualization by decreasing reverberant echoes, suppressing side and grating lobes, and reducing acoustic clutter in the images. In addition, the narrow beamwidth associated with the harmonic signal improves the lateral resolution of harmonic B-mode images relative to fundamental B-mode images [12], [13].

Coherence-based beamforming (e.g., short lag spatial coherence, or SLSC, imaging) has demonstrated remarkable improvements in thyroid imaging [14], lesion detection [15], endocardial border detection [16], [17], [18], fetal imaging [19], [20], liver imaging [21], and breast imaging [22], [23], [24]. SLSC imaging is implemented by computing the spatial correlation between received signals at various element separations (or lags), then summing across values calculated at these spatial lags to generate the final output image. Therefore, whereas traditional B-mode imaging relies on the magnitude of received signals, SLSC imaging relies on the spatial coherence between signals received by different transducer elements in the array.

Over the past decade, multiple variations of SLSC imaging have been developed and tested for various applications. Adaptation of SLSC to harmonic imaging has been shown to improve SLSC images due to decreased reverbration clutter in harmonic compared to fundamental signals [16], [25]. Therefore, harmonic SLSC imaging has the potential to improve contrast compared to fundamental SLSC images. To balance the higher spatial resolution incorporated in higher spatial lags with the improved contrast observed at lower spatial lags [26], Nair et al. [27] applied a weighting scheme (known as M-weighting) across different spatial lags prior to forming the final coherence image. Robust principal component analysis (RPCA) [28], [29] was additionally employed to decrease noise by rejecting coherence outliers. The combination of M-weighting with RPCA led to the development of the Robust SLSC (R-SLSC) beamformer, which offers improved contrast-to-noise ratio (CNR) and SNR when compared to SLSC images [27]. R-SLSC imaging can also be implemented without M-weighting, which we introduce and define as r-SLSC herein.

Our group recently introduced the first known demonstrations of coherence-based beamforming to distinguish solid from fluid breast masses [22], [23], [24]. In addition, Wiacek et al. [30] demonstrated that including R-SLSC images along-side B-mode images would have decreased the percentage of unnecessary biopsies from 43.3% to 13.3%, due to the improved diagnostic certainty of breast ultrasound images when assessed by five board-certified radiologists in a randomized reader study. Sharma et al. [31] and Kokumo et al. [32] combined harmonic imaging with R-SLSC imaging and SLSC imaging, respectively, to assess additional potential benefits of these combinations. Despite these advances, variations among radiologists were observed when diagnosing breast masses during the reader study noted above, highlighting the importance of reader-independent metrics to distinguish solid from fluid masses [23], [30], [33]. Contrast difference [22], [30], lag one coherence (LOC) [33], [34], coherence length [33], and generalized contrast to noise ratio (gCNR) [31], [35] were previously introduced and investigated as objective metrics for reader-independent distinction of solid and fluid breast masses.

Although multiple imaging modes and metrics have the potential to differentiate solid from fluid breast masses, these various demonstrations were performed with independent sets of breast data, which limit direct comparisons across studies [22], [23], [30], [31], [32]. A systematic, comparative investigation applied to a single dataset is required to determine the relative benefits and limitations. Therefore, the purpose of this article is to systematically compare the performance of eight coherence imaging modes relative to traditional fundamental and harmonic B-mode images to determine the best option to distinguish solid from fluid masses in breast ultrasound images from the same dataset. Extending our previous work, in which we compared fundamental and harmonic R-SLSC images of 18 masses and introduced the potential of gCNR as a reader-independent breast content classification metric [31], this article includes 40 masses and independently demonstrates the effects of harmonic imaging, M-weighting, and RPCA on coherence-based images to determine the most suitable beamformer to distinguish solid from fluid masses. In addition, previous work [30], [31], [33] independently identified contrast difference, LOC, and gCNR as the most promising metrics for the clinical task of distinguishing solid from fluid breast masses. These metrics are compared herein to determine the most suitable objective, reader-independent metric for clinical deployment.

The remainder of this article is organized as follows. Section II describes our methods and materials, including details about our patient population, data acquisition, beamforming techniques, and quantitative evaluation methods. Section III shares our results and their relevance to our study goals. Section IV discusses key insights based on our results, and Section V summarizes our major conclusions.

## II. METHODS AND MATERIALS

### A. Study Population

Thirty-one patients scheduled for ultrasound-guided aspiration or core needle biopsy of at least one breast mass were enrolled in our study. Patients ranged from 24 to 91 y in age, with a mean age of 55 y. Raw ultrasound radio-frequency (RF) channel data were acquired from these patients after receiving informed consent and approval from the Johns Hopkins Medicine Institutional Review Board (Protocol No. IRB00127110). Forty in vivo hypoechoic masses, including four incidentally noted simple cysts, were scanned and processed offline to form matched fundamental and harmonic B-mode and coherence images.

Simple cysts were classified as cysts without aspiration or biopsy because of clinical B-mode ultrasound features matching a simple cyst. Aspiration was performed on masses that appeared fluid in nature, and the masses that were successfully aspirated were designated as fluid masses. For the remaining masses, the pathology results of each core-needle biopsy served as the ground truth for mass classification.

The 40 masses total consisted of seven fluid-filled (four simple and three complicated cysts), five complex solid and fluid (hereafter referred to as mixed), and 28 solid (22 benign and 6 malignant) masses. Table I provides the pathology results and corresponding mass classification (i.e., simple cyst, complicated cyst, mixed solid and fluid, benign solid, malignant solid) of these 40 masses. The depth of each mass reported in Table I corresponds to the axial distance from the center of the mass to the surface of the ultrasound probe.

### B. Data Acquisition

An Alpinion ECUBE12R research ultrasound scanner (Alpinion, Seoul, South Korea) connected to a 128-element L8–17 probe with 64 receive elements, a center frequency of 12.5 MHz, and a sampling frequency of 40 MHz was employed to acquire raw ultrasound RF channel data with 256 receive scan lines per image. Each mass was insonified with a pulse-inversion harmonic imaging sequence, transmitted with a center frequency of 6 MHz. The focus of transmitted beams was located within 0–1 cm of the mass center, as selected by one of our board-certified radiologist coauthors (specialized in breast radiology) performing the scan (E.O., K.M., or E.A.). Fundamental channel data were formed with echoes received from the normal pulse, and harmonic channel data were formed with summed echoes from the normal and inverted pulses. The acquired fundamental and harmonic channel data were delayed offline to account for time-of-arrival differences prior to implementing additional beamforming then post-processing steps to display or evaluate the final results.

### C. Image Formation

#### 1) B-Mode Images:

Matched fundamental and harmonic B-mode images were created by applying a conventional delay-and-sum (DAS) beamformer to the fundamental and harmonic channel data, respectively, as follows:

(1) $$S_{DAS}=\sum_{i=1}^{N}s_{i}\left(n\right)$$

where $s_{i}(n)$ is the time-delayed, zero-mean signal received at element i from depth n, and N is the number of receive elements in the ultrasound probe. Equation (1) was repeated for each scan line, $S_{DAS}$, to form a single beamformed image. Each DAS-beamformed image was then envelope detected, normalized to its maximum value, and log compressed prior to being displayed as a B-mode image with 60-dB dynamic range.

#### 2) SLSC Images:

SLSC-based beamformers rely on the spatial coherence of delayed backscattered echoes received across the transducer aperture to form coherence-based images. Spatial coherence, $\overset{ˆ}{R}$, was calculated by normalizing the spatial covariance of time-delayed signals recorded by equally spaced elements (i.e., spatial lags) by the variance of each time-delayed signal [14]

(2) $$\overset{ˆ}{R}\left(m\right)=\frac{1}{N-m}\sum_{i=1}^{N-m}\frac{\sum_{n=n_{1}}^{n_{2}}s_{i}\left(n\right)s_{i+m}\left(n\right)}{\sqrt{\sum_{n=n_{1}}^{n_{2}}s_{i}^{2}\left(n\right)\sum_{n=n_{1}}^{n_{2}}s_{i+m}^{2}\left(n\right)}}$$

where m is the spatial lag (expressed as the number of element separations), and the axial correlation kernel, k, spans depths $n_{1}$ to $n_{2}$, centered on depth n. The value of the SLSC pixel was generated by summing the resulting spatial coherence function up to a specific short-lag value, M

(3) $$R_{sl}=\int_{1}^{M}\overset{ˆ}{R}\left(m\right)dm\approx \sum_{m=1}^{M}\overset{ˆ}{R}\left[m\right].\;$$

To create the final SLSC image, (2) and (3) were repeated in succession for each lateral and axial SLSC pixel position and all negative SLSC pixels were set to zero (based on the rationale that these small negative pixels adversely affect image quality and contrast measurements [27]).

#### 3) M-SLSC Images:

M-weighted SLSC (M-SLSC) images [27] were formed by applying a linearly decreasing weighting as a function of M to the SLSC images to incorporate higher lag values in the final images. At these higher lag values, the spatial resolution is generally improved, but the spatial coherence across the entire image (containing primarily tissue) generally decreases, which decreases the contrast of masses in the image. Therefore, the applied weights enable the inclusion of important boundary information provided by increased spatial resolution, without causing the resultant decrease in contrast typically observed when SLSC images are formed at higher spatial lags [26]. The M-SLSC pixel value was obtained as follows:

(4) $$R_{msl}=\sum_{m=1}^{M}\left(1-\frac{m-1}{M}\right)R_{sl[m]}\;$$

which corresponds to the linearly decreasing weightings of 1 and (1/M) at lags 1 and M, respectively. Negative pixels were set to zero prior to applying this weighting.

#### 4) r-SLSC Images:

RPCA was previously implemented to denoise SLSC images by taking advantage of the sparse, high spatial frequency information present at higher lags. This step was previously coupled with M-weighting [27], and we evaluate its impact independently by vectorizing each lag image (i.e., the SLSC images generated at each lag value up to lag value M) and stacking the vectors to create lag as the second dimension of a 2-D matrix. RPCA was implemented on this vectorized matrix, D, using the augmented Lagrangian multiplier (ALM) method [29], which solves for the minimum of the Lagrangian L(A,E,Y,μ) of the problem defined by

(5) $$L\left(A,E,Y,\mu \right)=∥A{∥}_{*}+\lambda ∥E{∥}_{1}+\;\left\langle Y,D-A-E\right\rangle \;\;+\frac{\mu }{2}∥D-A-E{∥}_{F}^{2\;}$$

where A is the desired low-rank ground truth matrix, E is a sparse error matrix, Y is a matrix of Lagrange multipliers, λ is the sparsity penalty parameter that can be varied to smooth tissue texture, μ is a positive scalar representing a reconstruction error equal to $1.25/∥D{∥}_{2}$ (as recommended in [29]), $∥\cdot {∥}_{*}$ is the nuclear norm, $∥\cdot {∥}_{1}$ is the $L_{1}$ norm, $∥\cdot {∥}_{F}$ is the Frobenius norm, and $∥\cdot {∥}_{2}$ is the dual norm of $∥\cdot {∥}_{*}$. After implementing RPCA (using the MATLAB inexact ALM solver based on [29] and hosted at [36]), the resulting denoised matrix A was summed across the lag dimension, vectorization was reversed, and the negative r-SLSC pixels were set to zero to yield the r-SLSC image, consisting of denoised pixel values, $R_{rsl}$.

#### 5) R-SLSC Images:

R-SLSC images [27] combine the RPCA step with the M-weighting step, resulting in images formed by applying the following equation to the r-SLSC pixel, $R_{rsl}$, as follows:

(6) $$R_{Rsl}=\sum_{m=1}^{M}\left(1-\frac{m-1}{M}\right)R_{rsl}\left[m\right].$$

#### 6) Coherence Parameter Selection and Post-Processing:

Coherence images typically include the short-lag region ranging between 1% and 30% of the receive aperture width [14]. To balance the trade-off between poor resolution and contrast at lower and higher M values, respectively [14], [26], fundamental and harmonic SLSC images were formed with *M* = 7, which corresponds to 10% of the receive aperture. A similar fixed approach to assigning M values in r-SLSC, R-SLSC, and M-SLSC images was implemented, which differs from the dynamic approach previously implemented to match tissue SNR (with M values ranging 15–30) for three reasons. First, tissue SNR does not provide information about mass contents, which is the focus of this article. Second, R-SLSC contrast (which does provide information about mass contents) was previously shown to be stable at higher lag values (e.g., ≥20) [22]. Third, the maximum possible M-SLSC, r-SLSC, and R-SLSC resolution exists when incorporating spatial lags as large as 30% of the receive aperture [22], [26]. Therefore, the fundamental and harmonic M-SLSC, r-SLSC, and R-SLSC images herein were formed with *M* = 20, which corresponds to 30% of the receive aperture width.

In simulations, Hyun et al. [37] and Bell et al. [26] showed that slightly increasing the kernel length beyond the typical value of one wavelength causes a linear increase in contrast and a minimal decrease in the axial resolution. Therefore, the coherence images were analyzed with a kernel length of five samples which corresponds to 1.56 times the wavelength associated with the center frequency of the ultrasound probe and is the same value used in our previous studies [22], [30]. In the r-SLSC and R-SLSC images, the sparsity parameter was *λ* = 1.

Each coherence image was normalized to its maximum value. To enhance the distinction between solid and fluid masses, the normalized coherence images were log compressed prior to being displayed with 60 dB dynamic range, as implemented in previous work [14], [22], [27]. This log compression enabled direct comparison with metrics reported in dB, while also presenting images with similar tissue brightness to B-mode images and simplifying the scaling process that would otherwise be necessary to achieve similar results with a linear display method [18], [21].

### D. Quantitative Evaluations

#### 1) ROI Selection:

Regions of interest (ROIs) within the mass and tissue were manually selected from fundamental B-mode images. First, an elliptical area within the mass was chosen as the mass ROI. Then, an elliptical region of the same size and at the same depth as the mass ROI, with its nearest edge located at a lateral distance of 0.9–8.2 mm from the nearest edge of the mass ROI, was chosen as the tissue ROI. In images with a mass that spanned the majority of the lateral field of view (nine masses total), the tissue ROI was selected from a tissue region at a depth <5 mm from the nearest edge of the mass ROI. For each mass, the same mass and tissue ROIs were implemented when calculating the following performance metrics for each beamforming method described in Section II–C.

#### 2) Contrast and Contrast Difference:

As contrast is an accepted and widely used metric in radiology [35], [38], [39], the contrast of each mass relative to its background tissue was computed to compare image quality and determine the ability of each beamforming technique to locate the mass. Contrast was measured as follows:

(7) $$\text{Contrast}\;=20\log_{10}\left(\frac{\mu_{\text{mass}\;}}{\mu_{\text{tissue}\;}}\right)$$

where $\mu_{\text{mass}\;}$ and $\mu_{\text{tissue}\;}$ are the mean beamformed signals (i.e., after envelope-detection for B-mode images, prior to normalization and log compression for B-mode and coherence images, with no dynamic range alterations that could affect contrast) within the mass and tissue ROIs, respectively. Based on previous reports [22], [30], contrast is expected to be higher in coherence-based images of fluid-filled masses when compared to amplitude-based images, whereas in solid masses, contrast is expected to be lower in coherence-based images when compared to amplitude-based images. Therefore, the difference between the contrast in B-mode and coherence images (i.e., contrast difference) is a possible metric to distinguish solid from fluid masses [22]

(8) $$\text{Contrast}\;\text{Difference}\;={\;\text{Contrast}\;}_{B\text{-mode}\;}-{\;\text{Contrast}\;}_{\text{Coherence}\;}\text{.}\;$$

The contrast difference of a fundamental coherence image was calculated by subtracting the contrast of the fundamental coherence image from the contrast of the corresponding fundamental B-mode image created from the same raw data. Similarly, the contrast difference of a harmonic coherence image was calculated by subtracting the contrast of the harmonic coherence image from that of the corresponding harmonic B-mode image created from the same raw data. As demonstrated in previous work [22], a positive contrast difference indicates that the mass is fluid, whereas a negative contrast difference indicates that the mass is solid.

#### 3) Lag One Coherence:

The mean coherence value within the mass ROI of a SLSC image created with lag M=1 is referred to as the mean LOC [33], where LOC is empirically determined by evaluating (2) at M=1 [34]


(9) $$LOC=\left\langle \frac{1}{N-1}\sum_{i=1}^{N-1}\frac{\sum_{n=n_{1}}^{n_{2}}\;s_{i}[n]s_{i+m}[n]}{\sqrt{\sum_{n=n_{1}}^{n_{2}}s_{i}^{2}[n]\sum_{n=n_{1}}^{n_{2}}s_{i+m}^{2}[n]}}\right\rangle .$$

The mean LOC was previously reported to achieve a sensitivity of 1 and a specificity of 1 when used as an objective metric to distinguish solid from fluid masses [33]. Therefore, the mean LOC inside the mass ROI of each fundamental and harmonic B-mode and coherence image was computed, using the same kernel length described in Section II–C6.

#### 4) gCNR:

We recently proposed gCNR as an alternative objective metric to distinguish solid from fluid masses [31]. Traditional image quality metrics like contrast, CNR, and SNR are unbounded and sensitive to image manipulation techniques like thresholding and dynamic range adjustments. Contrary to these traditional image quality metrics, the gCNR is a bounded metric resistant to dynamic range alterations and can be applied to multiple types of images, units, and scales [35], [40], [41], [42]. The gCNR of breast masses relative to surrounding tissue is measured as follows:

(10) $$gCNR=1-\sum_{j=1}^{𝒩}min\left\{h_{\text{mass}}\left(x_{j}\right),h_{\text{tissue}}\left(x_{j}\right)\right\}$$

where 𝒩 bins centered at $\left\{x_{1},x_{2},\ldots ,x_{𝒩}\right\}$ were defined to derive histograms $h_{\text{mass}\;}$ and $h_{\text{tissue}\;}$ of the beamformed signals (after envelope-detection for B-mode images; prior to normalization and log compression for B-mode and coherence images) of signals within the mass and the surrounding breast tissue ROIs, respectively, and j is the index of the bin. Although a fixed or data-driven approach to selecting 𝒩 can be used to compute the histograms [42], [43], [44], we relied on a previously reported approach [45] (validated in the Appendix of [42]) that uses the data-based method described by Wand [46] (resulting in 19 ≤ 𝒩 ≤ 304).

#### 5) Statistical Analysis:

Sensitivity and specificity are two common statistical metrics to evaluate and compare the performance of a classifier. The sensitivity and specificity of fluid mass detection were measured as the fraction of fluid masses correctly identified as fluid and the fraction of solid masses correctly identified as solid by the given metric, respectively,

(11) $$\text{Sensitivity}=\frac{\text{TP}}{\text{TP}+\text{FN}}$$

(12) $$\text{Specificity}=\frac{\text{TN}}{\text{TN}+\text{FP}}$$

where the definitions of true positive (TP), false negative (FN), true negative (TN), and false positive (FP) are based on previously reported mean LOC, contrast difference, and gCNR thresholds of 0.28 [33], 0 [30], and 0.73 [31], respectively, when evaluating each metric independently. More specifically, TP or FN was defined as a fluid mass with mean LOC below or above 0.28, respectively, contrast difference above or below 0, respectively, or gCNR above or below 0.73, respectively. Similarly, TN or FP was defined as a solid mass with mean LOC above or below 0.28, respectively, contrast difference below or above 0, respectively, or gCNR below or above 0.73, respectively. To avoid bias, we refrained from re-calculating thresholds for each metric, as the reported thresholds were previously calculated or determined using a different set of in vivo breast images to those presented herein. The LOC threshold was determined in [33] by plotting a receiver operating characteristic (ROC) curve for LOC values between −1 and 1, then finding the optimal threshold. The contrast difference threshold was chosen as 0 based on previous demonstrations [30] that a positive and negative contrast difference indicates that the mass is fluid and solid, respectively. The gCNR threshold was determined in [31] by applying a linear support vector machine to gCNR values of fundamental and harmonic R-SLSC images of solid and fluid breast masses.

#### 6) Signal Amplitude Analysis:

To assess the amplitude of harmonic relative to fundamental signals, the analysis described in [10] was implemented for each breast mass. In particular, regional variations in fundamental and harmonic images were displayed as contour maps after applying a low-pass filter to envelope-detected images (i.e., by convolving the envelope-detected RF data with a rectangular kernel of 78 × 15 pixels, which corresponds to 1.5 × 1.5 mm in the B-mode image). The pixel-wise ratio between the filtered fundamental and harmonic beamformed B-mode signals (after envelope-detection and prior to normalization and log compression) was calculated and discretized into 3 dB intervals ranging from −6 to 21 dB to display the contour map. The mean signal reduction within each contour map was also calculated.

## III. RESULTS

### A. Example Amplitude and Coherence Images

**Fig. 1.**
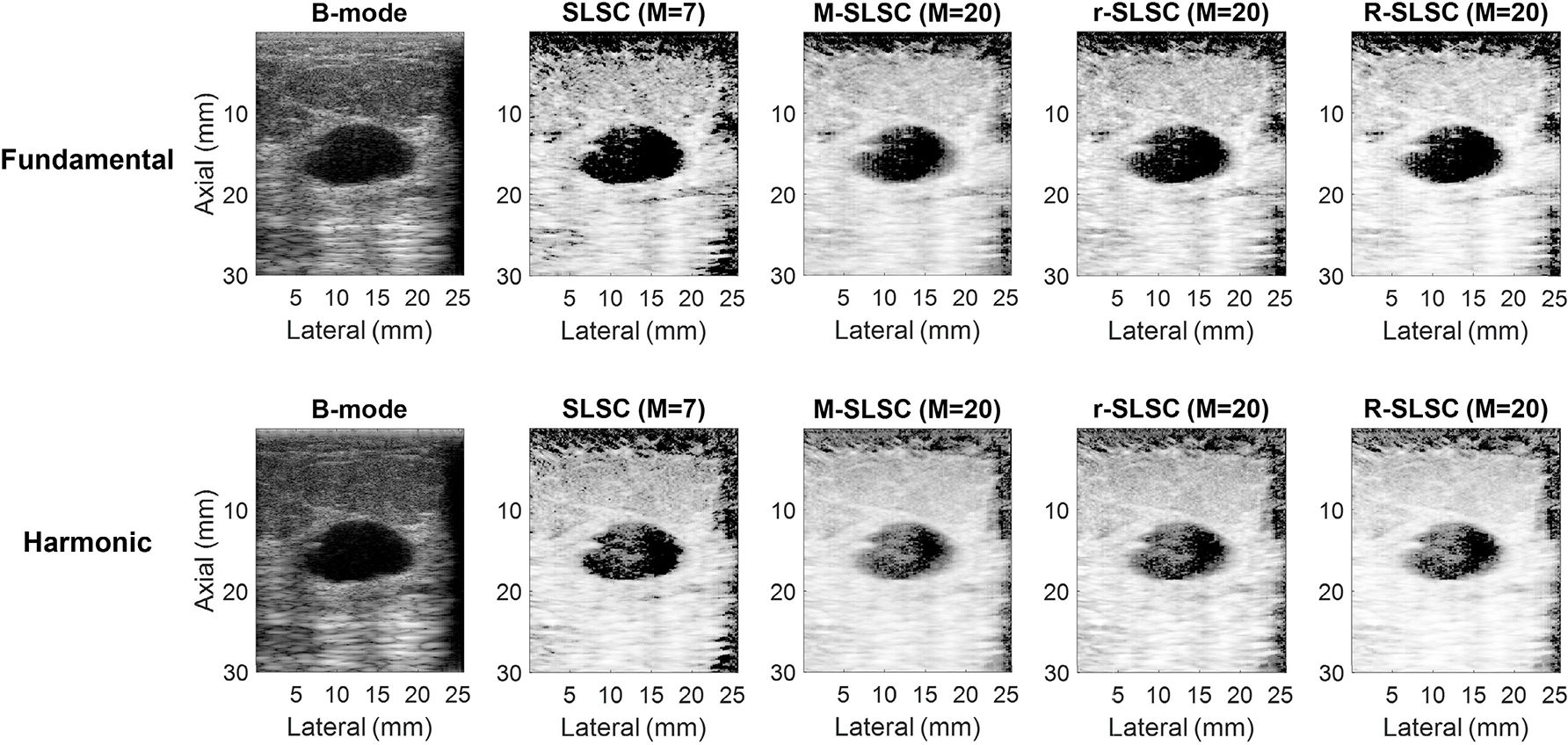
Fundamental and harmonic B-mode, SLSC, M-SLSC, r-SLSC, and R-SLSC images of a simple cyst (i.e., mass number 1 in Table I). Images are displayed with 60-dB dynamic range.

Fig. 1 shows fundamental and harmonic B-mode and coherence-based images of an example simple cystic mass. The mass can be distinguished from the background tissue in the ten images. When compared to the B-mode fundamental image, the B-mode harmonic image shows better boundary delineation, reduced acoustic clutter, and improved contrast. However, a decreased contrast in harmonic images (when compared to corresponding fundamental images) is not observed for the associated coherence-based images. When compared to the harmonic coherence-based images, the corresponding fundamental coherence-based images show better boundary delineation and improved contrast. In addition, SLSC fundamental and harmonic images have lower coherence inside the mass when compared to corresponding M-SLSC, r-SLSC, and R-SLSC images, demonstrating M-weighting and RPCA are responsible for the decreased visibility of this simple cyst.

**Fig. 2.**
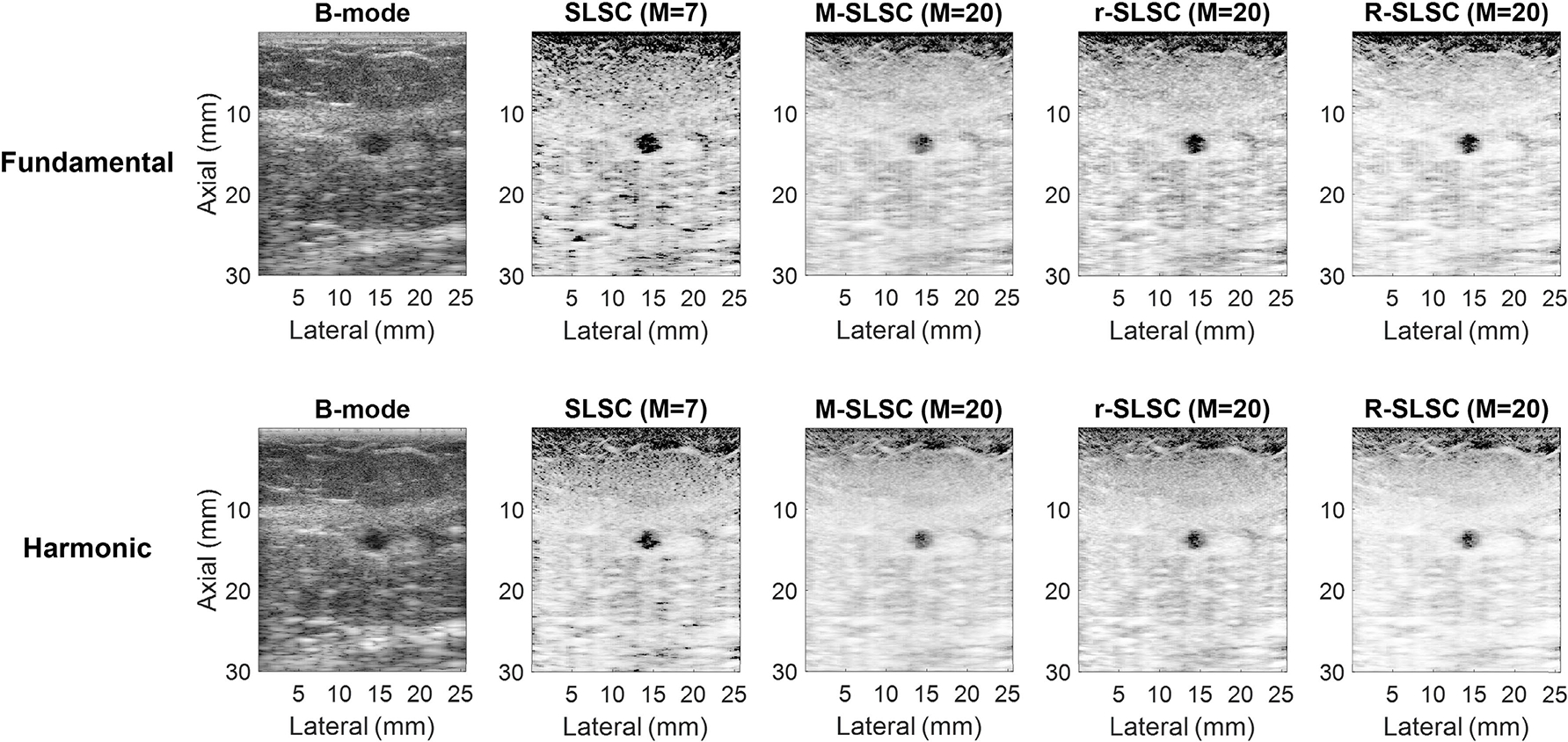
Fundamental and harmonic B-mode, SLSC, M-SLSC, r-SLSC, and R-SLSC images of a complicated cyst (i.e., mass number 6 in Table I). Images are displayed with 60-dB dynamic range.

Fig. 2 shows example images of a complicated cyst, which is generally distinguishable from the tissue background in fundamental and harmonic B-mode and coherence-based images. Qualitatively, the harmonic B-mode image provides better boundary delineation and improved contrast when compared to the fundamental B-mode image. However, fundamental SLSC, M-SLSC, r-SLSC, and R-SLSC qualitatively have better contrast when compared to matched harmonic images. In addition, the mass is more clearly distinguishable in fundamental and harmonic SLSC images when compared to fundamental and harmonic M-SLSC, r-SLSC, and R-SLSC images.

**Fig. 3.**
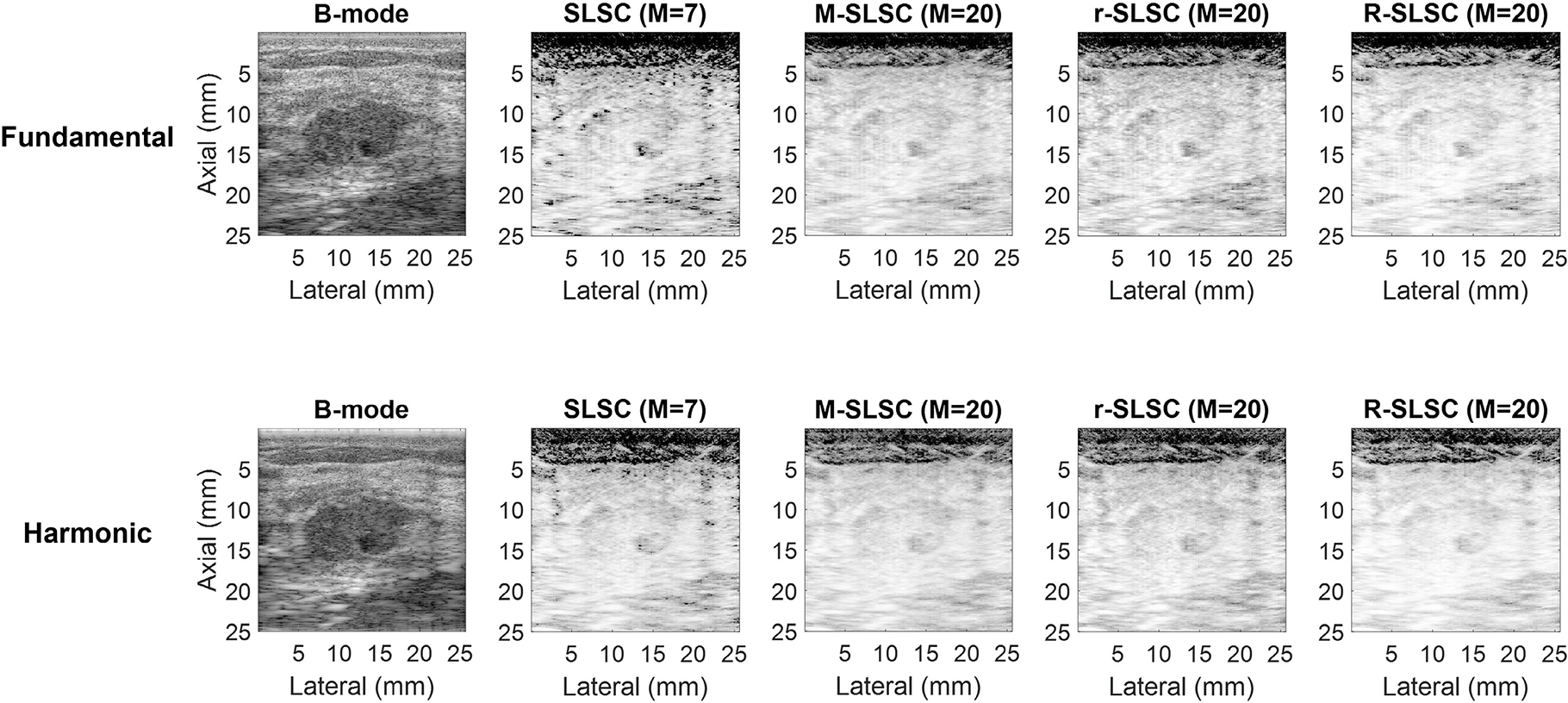
Fundamental and harmonic B-mode, SLSC, M-SLSC, r-SLSC, and R-SLSC images of a benign solid mass (i.e., a fibroadenoma with adenosis and cyst wall; mass number 16 in Table I). Images are displayed with 60-dB dynamic range.

Fig. 3 shows example images of a benign hypoechoic solid breast mass (i.e., a fibroadenoma with adenosis and cyst wall). Being hypoechoic with solid contents, the mass is visible in fundamental and harmonic B-mode images with high spatial coherence (similar to that of the surrounding tissue). Therefore, in the fundamental and harmonic coherence-based images, the mass blends with the background tissue and is less discernible when compared to the B-mode images.

**Fig. 4.**
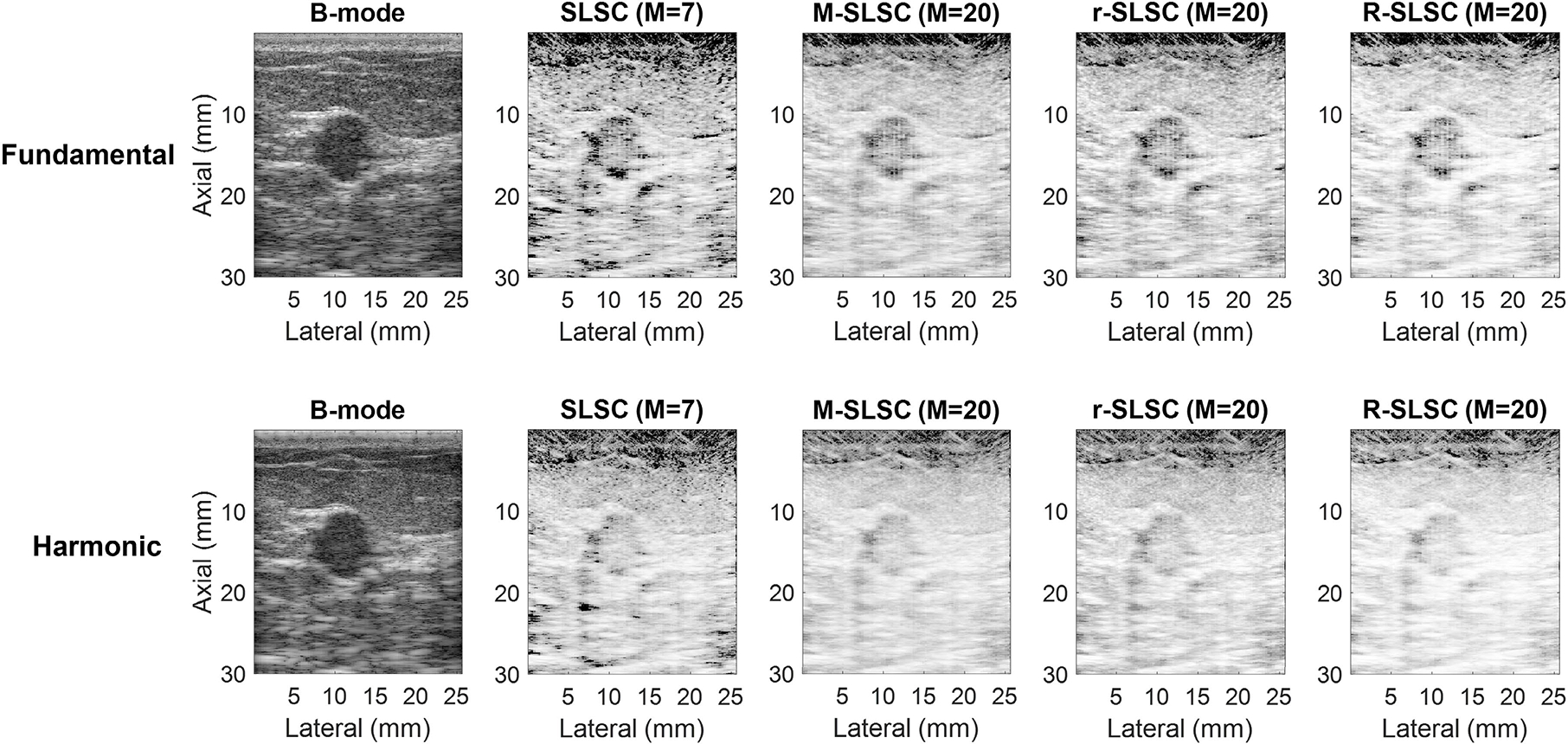
Fundamental and harmonic B-mode, SLSC, M-SLSC, r-SLSC, and R-SLSC images of a malignant solid mass (i.e., an invasive ductal carcinoma; mass number 36 in Table I). Images are displayed with 60-dB dynamic range.

Fig. 4 shows example images of a malignant solid mass (i.e., an invasive ductal carcinoma). Similar to Fig. 3, the mass is visible as a hypoechoic structure in fundamental and harmonic B-mode images and appears to be isocoherent with the background tissue in the coherence images. As a result, this solid mass is not clearly distinguishable from the surrounding tissue in fundamental and harmonic coherence images. In fundamental and harmonic SLSC images, dark regions are visible at the boundary of the mass (and in other areas of the tissue). These dark regions, representing areas of low spatial coherence and previously identified as artifacts [32], are more prominent in the fundamental coherence images when compared to matched harmonic coherence images. These dark-region artifacts are not unique to breast images, and they typically appear in coherence images when bright, coherent regions exist laterally to a less coherent region [18].

### B. Example Spatial Coherence Functions

**Fig. 5.**
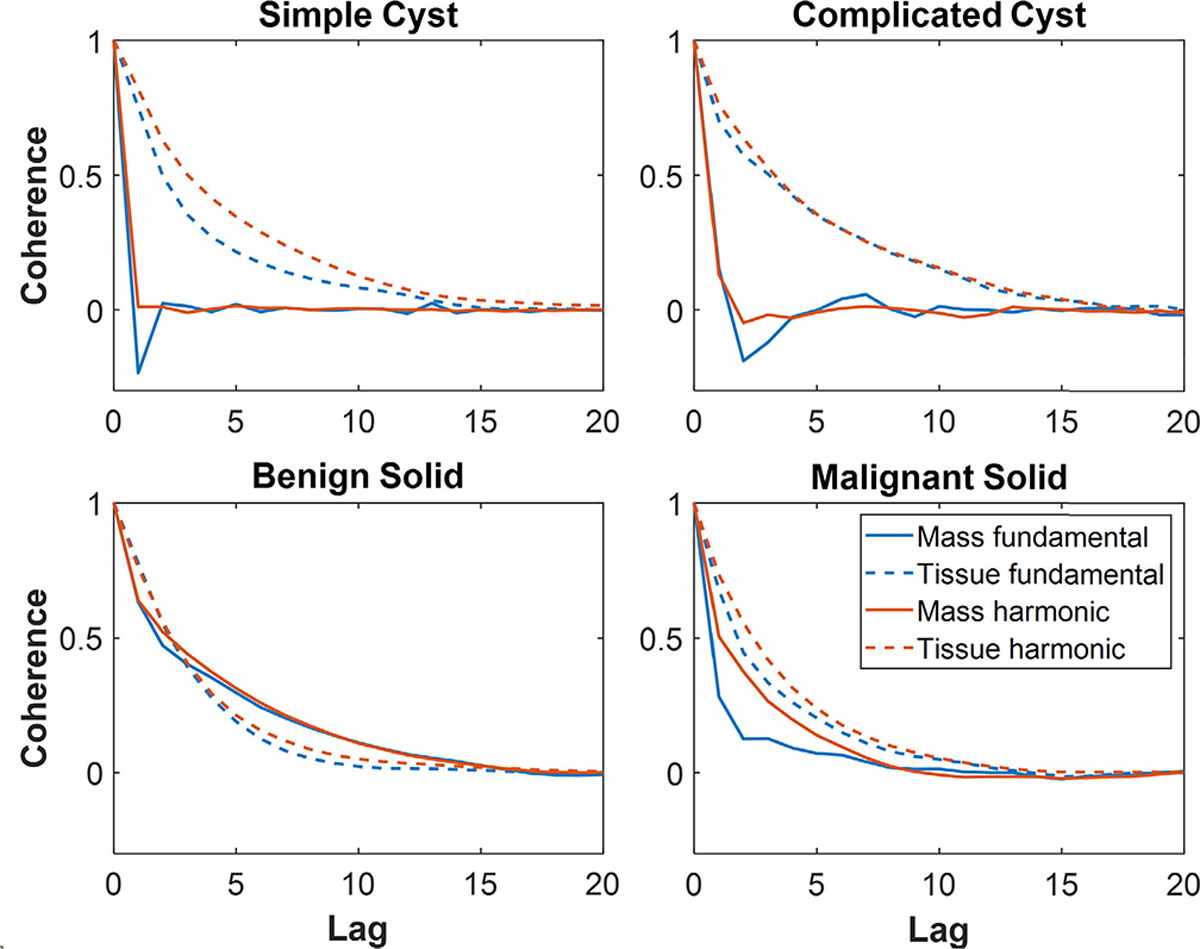
Mean coherence functions inside ROIs within masses and surrounding tissue for fundamental and harmonic images of the (a) simple cyst shown in Fig. 1, (b) complicated cyst shown in Fig. 2, (c) benign solid mass shown in Fig. 3, and (d) malignant solid mass shown in Fig. 4.

Fig. 5 shows the mean fundamental and harmonic spatial coherence functions [i.e., calculated with (2)], displayed within the short-lag region of selected ROIs (described in Section II–D1) within the mass and tissue regions of the example masses shown in Figs. 1–4. The spatial coherence within the simple and complicated cysts rapidly decreases as a function of spatial lag when compared to that of tissue in both fundamental and harmonic images. In the benign and malignant solid masses, the decrease in coherence within the mass as a function of spatial lag is similar to that of the tissue in both fundamental and harmonic images. These differences in coherence functions are responsible for cystic masses appearing darker than the background in coherence-based images and for solid masses appearing as isocoherent (i.e., blending with the tissue background in coherence-based images). When compared to fundamental coherence functions, the harmonic coherence functions in Fig. 5 generally have increased spatial coherence in both the mass and surrounding tissue. This increase in harmonic spatial coherence when compared to fundamental spatial coherence is more prominent inside the mass rather than within tissue in the simple and complicated cysts examples, particularly for lower spatial lags (i.e., *M* < 5). The observed increase in harmonic spatial coherence inside the simple and complicated cysts is responsible for the lower qualitative contrast of harmonic coherence-based images when compared to fundamental coherence-based images.

### C. Objective Assessment Metrics

**Fig. 6.**
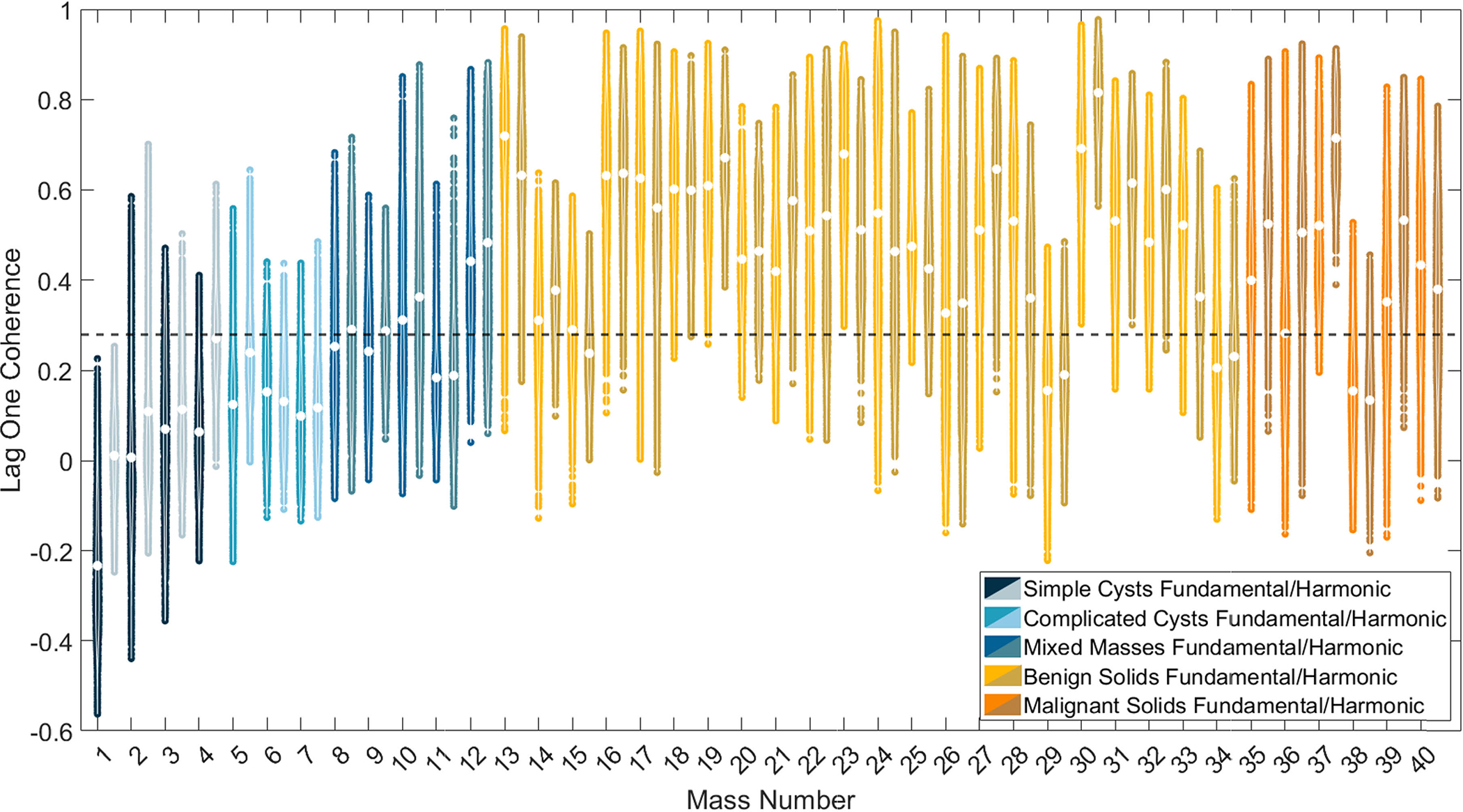
Violin plots of the distribution of LOC values of all pixels within each region of interest of each breast mass, labeled by the mass number reported in Table I. For each mass number, a pair of results from fundamental (left) and harmonic (right) data is reported. The white dot within each violin plot represents the mean LOC of the mass. The dashed horizontal line across the entire plot depicts the previously reported threshold (i.e., 0.28) to distinguish fluid from solid masses [33].

Fig. 6 shows violin plots of the LOC of the 40 breast masses imaged in this study. The white dot in each violin plot indicates the mean LOC inside the mass. The dashed line indicates the threshold reported in [33] to distinguish fluid from solid masses. The mean LOC of cystic masses is generally lower than that of solid masses and is also below the predetermined threshold indicated by the dashed line. The mean LOC was lower with fundamental compared to harmonic data in 6 out of 7 cystic masses. The mean LOC of mixed masses spanned both sides of the threshold and was lower in fundamental than harmonic images in each case. The mean LOC in fundamental and harmonic images of the solid masses was above the predetermined threshold in most cases.

Table II reports the LOC sensitivity and specificity of fluid mass detection based on the indicated threshold as 1 and 0.89, respectively, in fundamental data and 1 and 0.86, respectively, in harmonic data. Mixed masses were not considered when calculating the sensitivity and specificity as they generally contain both solid and fluid content. Among the 28 solid masses, the four masses incorrectly identified as containing fluid content were the shallowest of the masses (i.e., mass numbers 15, 29, 34, and 38 in Table I), highlighting one limitation of LOC as a metric to identify superficial solid masses (i.e., <5 mm from the transducer).

**Fig. 7.**
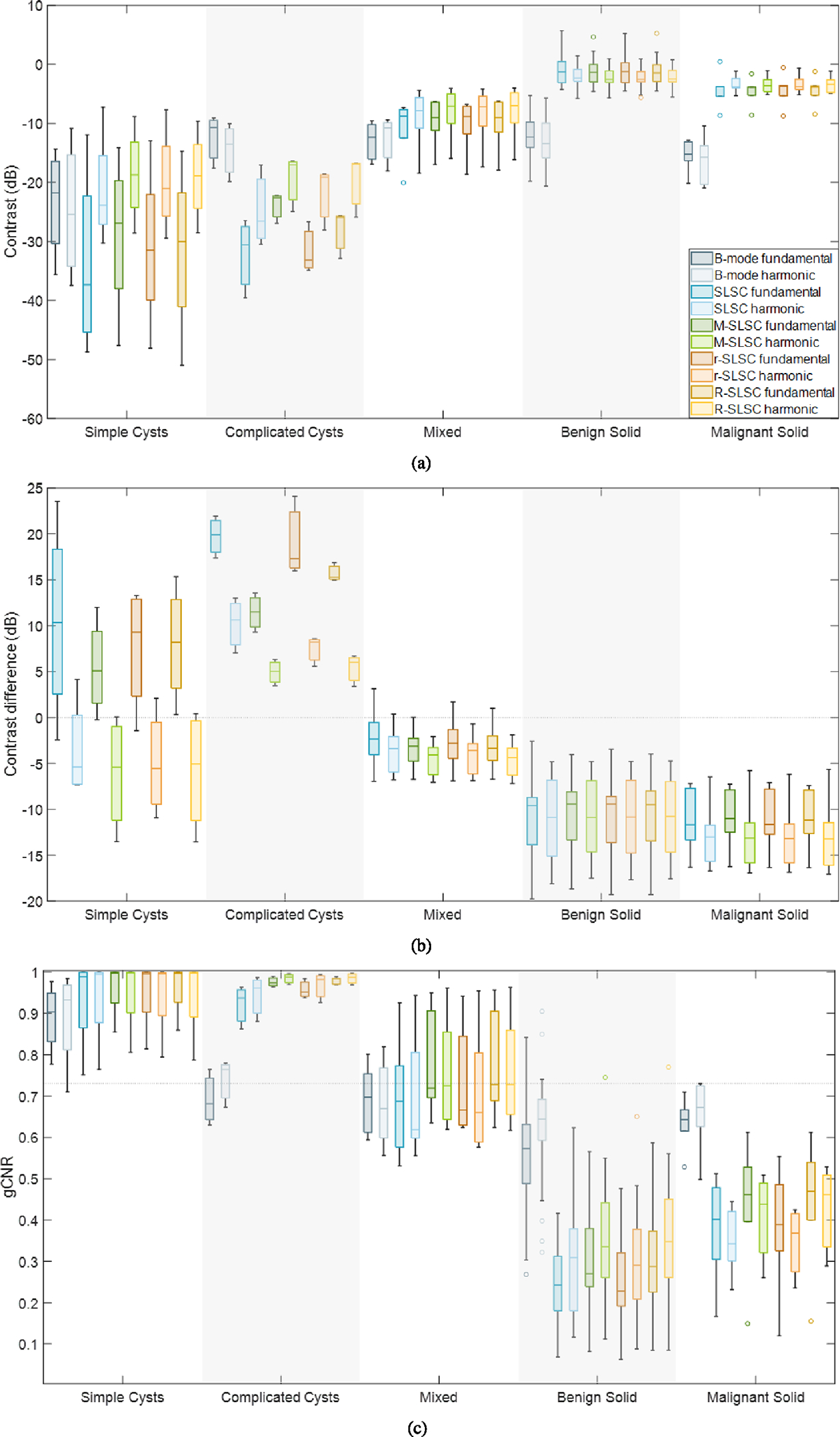
Box plots showing the distributions of objective metrics for the 40 breast masses reported in Table I, stratified by the five mass classifications reported in Table I. (a) Contrast in fundamental and harmonic B-mode, SLSC, M-SLSC, r-SLSC, and R-SLSC images. (b) Contrast differences between fundamental or harmonic B-mode images and corresponding SLSC, M-SLSC, r-SLSC, or R-SLSC images. (c) gCNR in fundamental and harmonic B-mode, SLSC, M-SLSC, r-SLSC, and R-SLSC images. The horizontal line within each box and the top-to-bottom box edges represent the median and interquartile range, respectively, per imaging mode per classification stratification. The vertical lines extending from each box represent the minimum and maximum values per imaging mode per classification stratification (excluding outliers, defined as values exceeding 1.5 times the interquartile range, which are represented as the circles). The dotted horizontal line extending across the plots in (b) and (c) shows the previously reported thresholds to distinguish fluid from solid masses (i.e., 0-dB contrast difference [30] and 0.73 gCNR [31], respectively).

Fig. 7(a) shows box plots summarizing contrast values for the five mass categories and ten imaging modes included in our study. The contrast was better (i.e., more negative) in simple cysts when compared to the fundamental and harmonic B-mode images of the other masses. Otherwise, the contrast of fundamental and harmonic B-mode images of complicated cysts, mixed solid and fluid masses, benign solid masses, and malignant solid masses were similar (i.e., range from −22 to −10 dB). Turning attention to the eight coherence imaging modes, the contrast of fundamental and harmonic coherence images range from −50 to −8 dB for simple and complicated cysts, with low negative or positive values (i.e., >−7 dB) for benign and malignant solid masses. For simple and complicated cysts, the contrast in fundamental SLSC, M-SLSC, r-SLSC, and R-SLSC images is better (i.e., more negative) than their harmonic counterparts. For mixed and solid masses, the contrast is similar in fundamental and harmonic SLSC, M-SLSC, r-SLSC, and R-SLSC images.

Fig. 7(b) shows the contrast difference [see (8)] measured for the 40 massess (grouped by mass category and stratified by fundamental and harmonic coherence imaging mode). The contrast difference is negative for benign and malignant solid masses and for most mixed masses, which agrees with qualitative observations that the contrast of hypoechoic masses with solid content is better with B-mode imaging when compared to coherence imaging (e.g., Figs. 3 and 4). Conversely, the contrast difference is consistently positive for the hypoechoic complicated cysts. This contrast difference distinction between solid and fluid masses generally persists for fundamental coherence images of simple cysts, for which the contrast difference is mostly positive. Otherwise, the contrast difference is mostly negative for the harmonic coherence images of simple cysts. The sensitivity and specificity of contrast difference to distinguish fluid from solid masses is reported in Table II. Of the eight coherence modes, only fundamental R-SLSC imaging yields a sensitivity and specificity of 1. Mixed masses were not considered for this analysis.

**Fig. 8.**
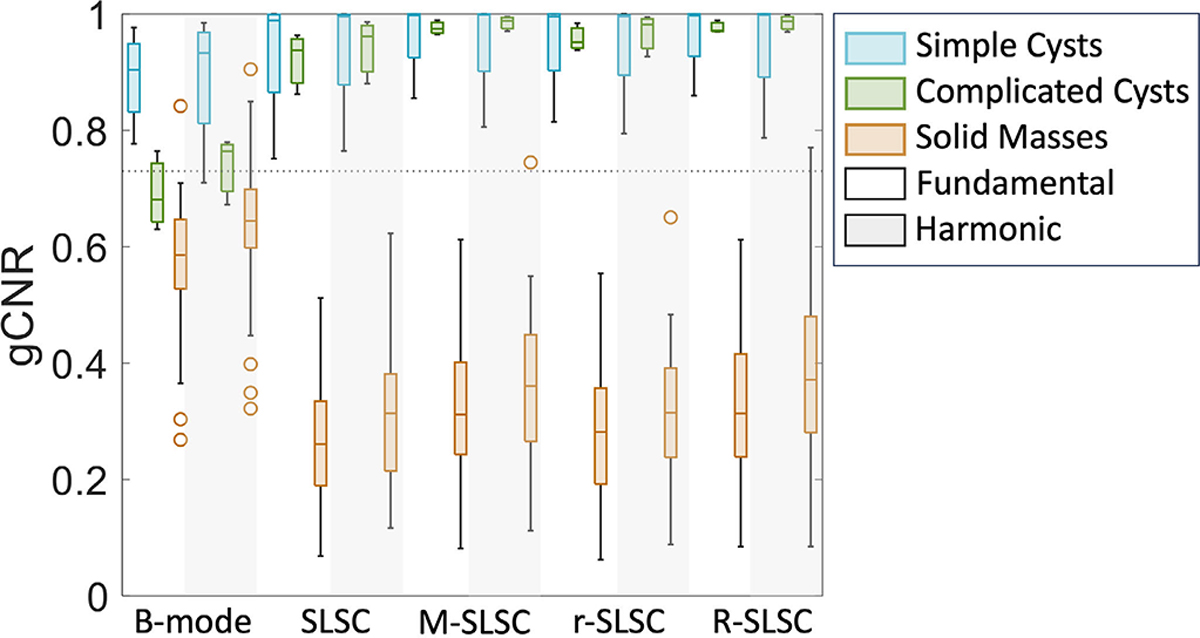
Box plots showing the gCNR distributions of the simple cysts, complicated cysts, and solid (benign and malignant) masses included in Fig. 7(c), as a function of imaging mode (i.e., fundamental or harmonic B-mode, SLSC, M-SLSC, r-SLSC, or R-SLSC). The dotted horizontal line shows the previously determined 0.73 gCNR threshold [31].

Fig. 7(c) shows box plots of the alternative gCNR metric, which is possible to deploy independently on coherence images alone (unlike contrast difference). This metric is reported for the same 40 masses stratified by the same mass categories and imaging modes as that in Fig. 7(a). In B-mode images, gCNR is generally higher for simple cysts (median of 0.90 and 0.93 in fundamental and harmonic images, respectively) when compared to the remaining mass types. Minimal variations in the range of gCNR values are also observed between fundamental and harmonic B-mode images for the same mass types, which is particularly true for complicated cysts. However, in the coherence images, gCNR is generally higher for simple and complicated cysts and lower for benign and malignant solid masses. Fundamental SLSC imaging best provides this distinction when considering the wider range of gCNR values (including outliers) across both benign and malignant solid masses obtained with other coherence imaging modes. To further demonstrate this optimal gCNR distinction provided by fundamental SLSC imaging, Fig. 8 consolidates the information in Fig. 7(c) to more directly compare the gCNR range of solid masses (benign and malignant) against the gCNR ranges of the fluid masses, as a function of imaging mode (with mixed masses excluded).

As reported in Table II, the sensitivity and specificity of gCNR to provide an objective, reader-independent metric to distinguish solid from fluid masses (with the predefined threshold of 0.73 [31]) is 1 and 1, respectively, for six of the eight coherence imaging modes (excluding mixed masses). This result is supported by the stark differences in gCNR values between fluid and solid masses observed in Figs. 7(c) and 8. For the remaining two coherence imaging modes (i.e., harmonic M-SLSC and R-SLSC imaging), although gCNR sensitivity remained as 1, gCNR specificity was reduced to 0.96, as reported in Table II, due to an outlier benign solid mass (i.e., mass number 33 in Table I). However, as observed in Figs. 7(c) and 8, a threshold that is determined independently for each coherence imaging mode will result in a gCNR specificity of 1 for the eight modes while maintaining a sensitivity of 1.

### D. Comparison of Fundamental and Harmonic Signal Amplitudes

**Fig. 9.**
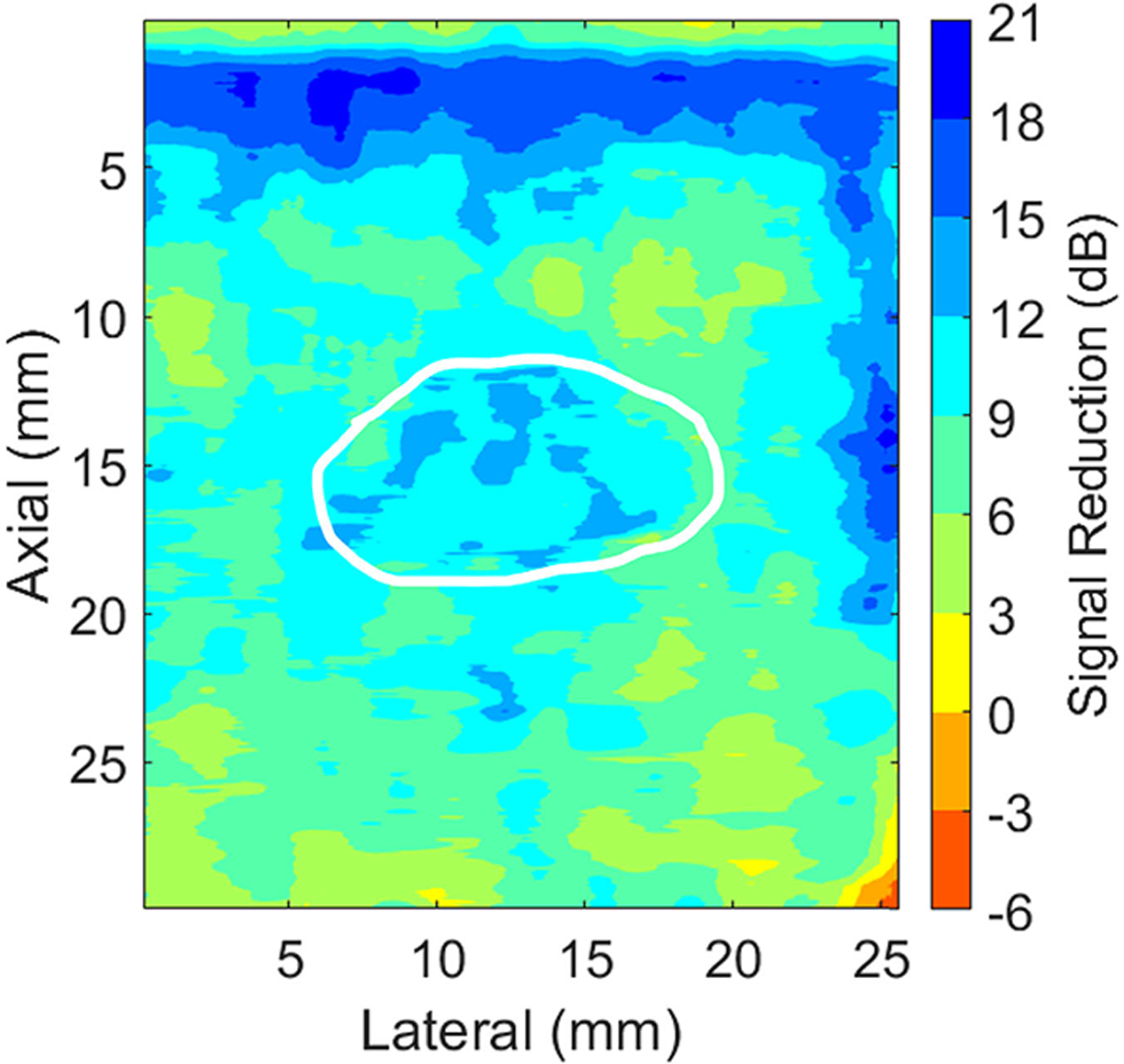
Contour plot of signal reductions in the harmonic relative to fundamental signals associated with the simple cyst images shown in Fig. 1. The white outline delineates the observed cyst boundary.

Fig. 9 shows a contour plot of the ratio of harmonic to fundamental signals associated with the example simple cyst shown in Fig. 1. There are regional harmonic amplitude signal reductions (e.g., greater reduction near the transducer surface and within the mass), with a mean reduction of 9.5 dB across the entire image. Across the 40 breast masses included in our study, the mean signal in harmonic images was 7.6–13 dB lower than that of fundamental images (mean ± one standard deviation reductions of 10.3 ± 1.4 dB), which is consistent with previous reports [10], [47]. In addition, there is qualitatively less acoustic clutter (e.g., Fig. 1) and quantitatively improved (i.e., more negative) contrast with harmonic B-mode images relative to fundamental B-mode images, particularly for complicated cysts, as reported in Fig. 7(a). These results collectively provide evidence that the harmonic imaging implementation utilized in our study produces amplitude results that are consistent with existing reports achieved with multiple ultrasound scanners [47], [48], [49].

## IV. DISCUSSION

This article is the first to demonstrate that multiple modes of fundamental and harmonic coherence-based images can successfully distinguish solid from fluid masses (relative to traditional fundamental and harmonic B-mode images), with two key insights. First, out of the eight coherence modes investigated, fundamental SLSC imaging generally offers the best qualitative and quantitative performance for the proposed task, particularly providing enhanced performance over the widely accepted harmonic B-mode imaging approach for complicated cysts (which represent a clinically problematic category of fluid-filled masses [50]). Therefore, previous indications that unnecessary procedures for fluid breast masses (e.g., complicated cysts) can be reduced with R-SLSC imaging [30] are now supported by new evidence pointing to fundamental SLSC imaging as the best option to maximize this clinical impact. Second, to address known challenges with associated reader variability [23], [30], [33], our comparison of promising objective metrics (i.e., LOC, contrast, contrast difference, and gCNR) highlight gCNR as the most optimal choice. This insight is based on quantitative comparisons of the same linear (rather than log-compressed) data, with no additional image manipulation implemented or required.

Although harmonic B-mode images are known to decrease clutter and improve contrast when compared to fundamental B-mode images [11] [also supported by Figs. 1, 2, and 7(a)], the coherence functions in Fig. 5 reveal increased coherence in fluid masses in harmonic images when compared to fundamental images. This increased coherence likely occurs because of the reduced incoherent clutter inside fluid masses (see Fig. 9), resulting in reduced coherence differences between fluid masses and surrounding tissue, leading to worse contrast in harmonic relative to fundamental coherence images, as observed qualitatively (Figs. 1 and 2) and quantitatively [Figs. 6 and 7(a)]. Images of simple and complicated cysts (Figs. 1 and 2, respectively) demonstrate improved mass detection and boundary delineation in fundamental SLSC, M-SLSC, r-SLSC, and R-SLSC images when compared to their harmonic image counterparts. Similarly, the lower LOC (Fig. 6) and improved contrast [Fig. 7(a)] in fundamental coherence images of fluid masses contributes to better discrimination of these masses from solid masses (when compared to harmonic coherence images). Therefore, we provide new and foundational empirical evidence to demonstrate both qualitatively and quantitatively that fundamental coherence imaging is better than harmonic coherence imaging when detecting fluid mass contents.

The conclusion that fluid masses have lower spatial coherence with fundamental relative to harmonic SLSC imaging is quantitatively supported by Figs. 5–7(a) and by the following additional evidence. First, similarly lower fundamental relative to harmonic spatial coherence functions were achieved in earlier reports (see Fig. 5 in [25]). Second, we achieved expected harmonic imaging benefits (Section III–D) and associated signal amplitude reductions (Fig. 9), indicating that our conclusions are not limited to our specific ultrasound scanner. Third, given that nonlinear acoustic propagation is underdeveloped in the near-field region of the transducer [51], with additional consideration that the depth of a breast mass (e.g., 1–2 cm) is generally less than that of a fetus or liver, the positive effects of harmonic SLSC imaging compared to fundamental SLSC imaging seem to be more prominent in fetal [19], [52] and liver [25] imaging relative to breast imaging. However, the contrast gains with in vivo harmonic SLSC liver imaging were marginal (e.g., 0.1 dB increase from 8.9 to 9 dB) or worse (e.g., 2 dB mean decrease from 11.08 dB to 9.18), when compared to corresponding fundamental SLSC images [21]. This combined evidence supports the superiority of fundamental SLSC imaging (relative to harmonic amplitude and coherence imaging) for the proposed clinical task.

From a clinical perspective, traditional B-mode imaging is generally suitable for identifying simple cysts [4], [38], which is supported by the gCNR results in Fig. 7(c). The negative contrast differences measured from the harmonic coherence images of simple cysts [Fig. 7(b)] may seem unexpected and counterintuitive given that the identification of simple cysts is not a clinical challenge. However, this observation is supported by the generally better (i.e., quantitatively more negative) contrast of fundamental coherence images relative to fundamental B-mode images and the generally worse (i.e., quantitatively less negative) contrast of harmonic coherence-based images when compared to harmonic B-mode images of simple cysts, as shown quantitatively in Fig. 7(a) and qualitatively in Fig. 1.

As opposed to the easily identifiable anechoic simple cysts, complicated cysts are typically hypoechoic in comparison, causing increased difficulty for radiologists to confidently distinguish complicated cysts from masses containing solid content with B-mode images alone [38], [50], [53], [54]. As a result, complicated cysts are currently among the most difficult to distinguish with fundamental or harmonic B-mode imaging alone, necessitating additional procedures or follow-up visits to diminish uncertainty [50]. This difficulty and uncertainty is highlighted with the contrast measurements in Fig. 7(a), given the contrast similarity among fundamental and harmonic B-mode images of complicated cysts, mixed, and solid masses. Evidence of this difficulty is also present (albeit to a lesser extent) among the B-mode gCNR results in Figs. 7(c) and 8. The improved contrast of complicated cysts qualitatively (Fig. 2) and quantitatively [Fig. 7(a)] achieved with fundamental and harmonic coherence imaging when compared to corresponding B-mode images is supported by the consistently positive contrast difference for complicated cysts [Fig. 7(b)], whereas consistently negative contrast differences were obtained for solid masses. Note that these consistencies were achieved with direct quantitative contrast comparison across image domains without implementing histogram matching recommendations [44], [55], which are not always feasible to implement [56]. Aside from the generally low sensitivity reported in Table II (with the exception of fundamental R-SLSC imaging), one drawback of the contrast difference metric is the requirement to beamform both B-mode and coherence images, followed by the required selection of both mass and background ROIs for analysis of both the B-mode and coherence images (whereas LOC only requires a coherence computation and a single ROI selection within an indeterminate mass and gCNR only requires analysis of the coherence image).

Given the limitations of contrast difference noted above and in Table II, when considering the most suitable alternative objective, reader-independent metric to discriminate mass contents, there are multiple trade-offs between LOC and gCNR. First, LOC provides the advantage of only requiring users to identify the mass ROI. When using LOC and the associated threshold that was previously determined [33], fluid masses were correctly identified as fluid (i.e., specificity of 1). However, among the 28 solid masses imaged, four masses were incorrectly identified as fluid in harmonic images and 3 out of those 4 were incorrectly identified as fluid in fundamental images. A deeper analysis revealed that these four masses were present at a depth of <5 mm from the skin surface, which is likely responsible for the lower LOC, as spatial coherence is generally lower in the near-field region [14], [33]. Therefore, the specificity of LOC was 0.89 for fundamental and 0.86 for harmonic imaging, as reported in Table II. It is promising that the sensitivity and specificity of gCNR outperformed both LOC applied to fundamental and harmonic data and contrast difference applied to fundamental and harmonic SLSC images. However, gCNR has the disadvantage over LOC of requiring users to identify the background ROI along with the mass ROI. Traditional or deep-learning-based image segmentation methods [57] have the potential to assist with ROI selection if a more automated approach is desired.

In addition to considering objective performance, the practical considerations of computational implementation further dictate preferences among the proposed methods. For example, RPCA is a computationally expensive step, and the results in Figs. 6 and 7(a) indicate that the required computational power and time to obtain r-SLSC and R-SLSC images outweigh the qualitative benefits shown in Figs. 1–4 (notwithstanding additional parameter optimization that can potentially be implemented to further improve results [22], [27]). Aside from the reduction of dark region artifacts, it is otherwise not clear that M-weighting offers the originally anticipated benefits, either when combined with RPCA or when implemented independently of RPCA, based on the generally similar performance between M-SLSC and R-SLSC images in Fig. 7 (e.g., similar gCNR in complicated cysts, similar contrast and gCNR with the remaining masses). These observations provide additional support for fundamental SLSC imaging as the preferred choice to distinguish solid from fluid masses (among the eight coherence imaging modes compared in this work). Therefore, our future clinical development of this application will be dedicated to the fundamental SLSC imaging approach, including novel deep learning [58] and real-time [59] solutions.

One limitation of our study is the focus on fluid versus solid mass distinction. However, there is some indication in Fig. 7(c) that gCNR combined with fundamental or harmonic M-SLSC or R-SLSC imaging has the potential to objectively distinguish complicated cysts from mixed solid and fluid masses, which is another clinically challenging task with B-mode imaging alone [53]. In addition, we understand that the ability to distinguish malignant from benign (solid or mixed) breast masses will provide additional clinical value. However, our coherence-based approach to address unnecessary biopsies of previously uncertain fluid breast mass contents has the potential to redirect clinical resources and future engineering efforts to improve the breast cancer detection process overall. In addition, although breast masses were presented and compared in this study, coherence-based imaging has the potential to distinguish solid from fluid content in suspicious masses found in other organs (e.g., liver [60], [61], pancreatic [62], or testicular [63] masses). Therefore, the results of our study are likely applicable to areas that extend well beyond breast imaging.

## V. CONCLUSION

This article presents a comparative, systematic study of fundamental and harmonic coherence-based imaging methods previously reported to distinguish fluid from solid breast mass contents. Eight coherence imaging modes (i.e., fundamental and harmonic SLSC, M-SLSC, r-SLSC, and R-SLSC imaging) were investigated qualitatively and quantitatively with four objective metrics (i.e., LOC, contrast, contrast difference, and gCNR) to provide a comprehensive summary of the advantages and limitations of these various metrics and imaging modes, with fundamental and harmonic B-mode images as the baseline. Results indicate that SLSC imaging is better at distinguishing fluid masses than M-SLSC, r-SLSC, and R-SLSC due to better contrast and fewer processing steps required (which inevitably reduces computational complexity and associated processing times). In addition, fundamental SLSC imaging was determined to be more suitable than harmonic SLSC imaging due to the lower coherence and better contrast of fluid masses (i.e., simple and complicated cysts), which is particularly true relative to solid or mixed masses. As an objective metric of fluid versus solid mass contents for potential for reader-independent mass evaluation, gCNR generally provided the greatest sensitivity and specificity, relative to LOC, contrast, and contrast difference. These insights establish a clinical path forward to improve the diagnostic certainty of breast mass contents at the time of an initial ultrasound exam.

## Figures and Tables

**TABLE I:** Pathology Results, Classification, and Axial Distance Between the Center of the Mass ROI and the Ultrasound Probe for the 40 Masses Investigated

Mass Number	Pathology Result	Classification	Mass Depth (mm)
1	Simple cyst	Simple Cyst	15.3
2	Simple Cyst	Simple Cyst	9.2
3	Simple Cyst	Simple Cyst	13.5
4	Simple Cyst	Simple Cyst	19.8
5	Complicated cyst	Complicated Cyst	18.5
6	Complicated cyst	Complicated Cyst	13.8
7	Complicated cyst	Complicated Cyst	9.6
8	Fat necrosis	Mixed	6
9	Cyst wall with chronic inflammation	Mixed	5.2
10	Clustered apocrine cysts	Mixed	7.7
11	Fibrocystic changes, apocrine metaplasia, PASH, and UDH	Mixed	10.1
12	Fibrocystic changes and duct hyperplasia	Mixed	10.5
13	Fibroepithelial lesion	Benign Solid	12
14	Fibroadenoma and sclerosing adenosis	Benign Solid	7.5
15	Stromal fibrosis	Benign Solid	3.4
16	Fibroadenoma with adenosis and cyst wall	Benign Solid	12.5
17	Fibroadenoma with stromal pseudoangiomotous	Benign Solid	10
18	Dense stromal fibrosis and fibroadenomatoid changes	Benign Solid	20
19	Pseudoangiomatous stromal hyperplasia and fibrocystic changes	Benign Solid	9.8
20	Sclerosing adenosis	Benign Solid	10.6
21	Cyst wall with stromal fibrosis	Benign Solid	9.5
22	Fibroadenoma	Benign Solid	8.77
23	Pseudoangiomatous stromal hyperplasia and fibroadenomatous change	Benign Solid	5.7
24	Fat necrosis with dense scar and focal microcalcification	Benign Solid	6.5
25	Reactive lymphoid hyperplasia	Benign Solid	8.7
26	Fibroadenoma	Benign Solid	5.7
27	Fibroadenoma	Benign Solid	10.7
28	Clusters of cysts with associated papillary apocrine metaplasia	Benign Solid	6.7
29	Fibroadipose tissue with collagen fibrosis	Benign Solid	3.5
30	Fibroadenoma	Benign Solid	17
31	Stromal fibrosis	Benign Solid	11.4
32	Stromal fibrosis	Benign Solid	9
33	Cyst wall with stromal fibrosis	Benign Solid	5
34	Abundant hemorrhage and fibrin	Benign Solid	4.4
35	Papillary carcinoma	Malignant Solid	10.5
36	Invasive ductal carcinoma	Malignant Solid	14
37	Invasive ductal carcinoma	Malignant Solid	18.8
38	Invasive ductal carcinoma	Malignant Solid	2.9
39	Invasive ductal carcinoma	Malignant Solid	11.5
40	Invasive ductal carcinoma	Malignant Solid	15.1

**TABLE II:** Sensitivity and Specificity of Mean LOC, Contrast Difference, and gCNR to Identify Fluid Mass Contents in Fundamental and Harmonic Data and in Fundamental and Harmonic SLSC, M-SLSC, r-SLSC, and R-SLSC Images. Mixed Solid and Fluid Masses Were Not Considered When Calculating Sensitivity and Specificity

		Fundamental				Harmonic			

**LOC**	Sensitivity	1				1			
	Specificity	0.89				0.86			

		SLSC	M-SLSC	r-SLSC	R-SLSC	SLSC	M-SLSC	r-SLSC	R-SLSC

**Contrast Difference**	Sensitivity	0.86	0.86	0.86	1	0.57	0.57	0.57	0.57
	Specificity	1	1	1	1	1	1	1	1
**gCNR**	Sensitivity	1	1	1	1	1	1	1	1
	Specificity	1	1	1	1	1	0.96	1	0.96

## Highlights

- Fundamental and harmonic B-mode, SLSC, M-SLSC, r-SLSC, and R-SLSC imaging of breast masses and four objective metrics to distinguish solid from fluid contents were comprehensively compared.
- Fundamental SLSC imaging generally has the best qualitative and quantitative performance with enhanced performance over harmonic B-mode imaging when distinguishing complicated cysts from solid masses.
- As an objective metric for potential reader-independent mass content evaluation, gCNR generally provided the greatest sensitivity and specificity, relative to LOC, contrast, and contrast difference.
